# The Coach–Athlete Relationship in Strength and Conditioning: High Performance Athletes’ Perceptions

**DOI:** 10.3390/sports7120244

**Published:** 2019-12-04

**Authors:** Steven J. Foulds, Samantha M. Hoffmann, Kris Hinck, Fraser Carson

**Affiliations:** Centre for Sport Research, School of Exercise and Nutrition Sciences, Deakin University, Geelong, VIC 3220, Australia; s.foulds@deakin.edu.au (S.J.F.); s.hoffmann@deakin.edu.au (S.M.H.); kris.hinck@deakin.edu.au (K.H.)

**Keywords:** leadership, coach behaviour, strength and conditioning coaching, coach effectiveness

## Abstract

The primary purpose of this study was to investigate high performance athlete perceptions of strength and conditioning coaches, specifically, (1) their character traits, (2) the effective behaviours that display these traits, and (3) how these relationships were fostered. Using the 3+1 C’s model of coach–athlete relationships as a framework (Jowett, 2007), 12 semi-structured interviews were conducted with high performance athletes (six female; six male) representing a variety of sports (i.e., freestyle wrestling, triathlon, field hockey, cycling, rowing, rugby union, netball, table tennis, and ice hockey). Participants ranged in age from 18 to 53 years (M = 29, SD = 9). Interviews took between 19–47 min and were transcribed verbatim. The transcripts equated to a total of 188 pages of data that were analyzed, coded, and further grouped into higher-order themes and general dimensions. The findings revealed 14 higher-order themes categorized under the 3+1 C’s general dimensions of closeness, commitment, complementarity, and co-orientation.

## 1. Introduction

Previous research has shown sports coaches have the ability to significantly influence athletes through their behaviours, communicative actions, and environments they create [1]. A positive coach–athlete relationship is acknowledged to promote participation, athlete satisfaction, self-esteem, and improved performance [2,3]. However, little research on coach-leadership has been conducted in the specific context of strength and conditioning (S&C) settings. 

Leadership is the behavioural, psychological, and social process of influencing others to move towards specific objectives [4]. To be effective, an adaptable leadership style must be developed, which will vary depending on the individual, the situation, and the needs of followers [5]. Leadership effectiveness is enhanced by applying consistent integration of professional, interpersonal, and intrapersonal knowledge with the intent to improve an individual’s competence, confidence, connection, and character [6].

Within sport, leadership research has focused on the coach–athlete relationship [7,8]. Jowett and Poczwardowski [2] defined the coach–athlete relationship as “a situation in which a coach’s and an athlete’s cognitions, feelings and behaviours are mutually and causally interrelated” (p. 4). The importance and motivation for studying the coach–athlete interpersonal dynamic lies in its practical applications, providing opportunities for coaches to be more effective when managing their interpersonal exchanges [8].

Since the 1970s three main models of leadership have been presented within sports coaching literature: (1) Multidimensional model of leadership (MDML) [9]; (2) Mediational model of leadership (MML) [10]; and (3) 3+1 C’s conceptual model [11]. Chelladurai [9] developed the MDML as a framework for effective leadership behaviours in specific sports situations, with athlete performance and satisfaction being viewed as products of the interaction of leadership. The MDML identifies a successful coach–athlete relationship as one where there is a congruence between the coaching behaviour preferred by the athlete, actual coaching behaviours displayed by the coach, and the situational requirements. It was recommended that the closer the coach can align their behaviour to these preferences, the greater the chance of athlete satisfaction and positive athletic outcomes. 

The MML was created as a tool for assessing and developing coaches’ behaviour and considers the relationship between coach behaviour, athlete perception, and athlete response [10]. It suggests that these behaviours, perceptions, and responses are a result of individual coach, athlete, and situational factors, and the effectiveness of different communication styles is dependent on each individual. For example, if an athlete perceives coaching behaviour as positive and supportive, then she or he is more likely to react in a positive and cooperative manner (or vice-versa).

The MDML and MML suggest that the coach–athlete relationship is unidirectional, with the coach leading the process. Jowett and Cockerill [12] postulated that the coach–athlete relationship is actually dyadic, with both the coach and athlete influencing it. Subsequently, Jowett [13] developed the “3 C’s” model to measure this dyad. The “3 C’s” included; (1) ‘closeness’: The depth of how the coach and athlete are connected and how trust, like, respect, and appreciation are expressed; (2) ‘commitment’: The desire to maintain the relationship over time and; (3) ‘complementarity’: The interaction between the coach and the athlete that is perceived to be cooperative and effective. Later, ‘co-orientation’ was added to assess how reciprocal the coach and athlete perceptions of the relationship were. The addition of ‘co-orientation’ resulted in the model being currently referred to as the “3+1 C’s” model [11]. The “3+1 C’s” model suggests the more an athlete and coach are satisfied with the relationship between them, the higher the quality of the relationship, and the greater the athletic outcomes [14]. Further, research indicates when the coach–athlete relationship was close and positive, athletes showed a desire to perform better [3]. Commitment to a “shared purpose” instils belief success can be achieved together [15], while longer relationships have been shown to have higher member satisfaction [16]. Complementarity from the coach can be developed by continuously analyzing athlete’s response to their behaviour, ensuring it complements the athlete’s requirements and is adjusted accordingly to achieve the desired outcome [17], while co-orientation is defined as the degree to which an athlete and coach are able to accurately infer how his or her coach or athlete is feeling, thinking, and behaving [2].

The primary aim of this project was to investigate high performance (i.e., participating at Olympic or Paralympic, professional, or state-, or national-, or international-level) athletes’ perceptions of S&C coaches, specifically, (1) their character traits, (2) their effective behaviours that display these traits, and (3) how these relationships are fostered. While there is a wealth of research on coach–athlete relationships in general sports coaching, limited research has been conducted specific to S&C coaches [18]. There are many important differences between the sports coach–athlete relationship, and the relationship between an S&C coach and their athlete. Commonly, the S&C coach has greater opportunities to create small groups and one-on-one situations to mutual goal set, show progress and have conversation beyond sport, particularly during return-to-play from injury periods. In addition, it has been previously reported athletes appreciate the importance of S&C coaches in achieving athletic success [19], however, it is not currently known how these relationships are developed initially and sustained over time. This knowledge will provide insight that may assist with the development of transferable skills amongst S&C coaches to complement their technical skills. 

## 2. Materials and Methods

### 2.1. Participants

Following institutional ethics approval (HEAG-H 108_2018), 12 high performance athletes (6 females and 6 males; mean ± SD age, 29 ± 9 years), ranging from state level representatives to Olympic medalists, from a range of sports were interviewed. While recognising that the S&C coach will have a slightly different role dependent on the sport, the S&C coach has a specific focus to maximize the physical condition of each athlete for performance. Participants were all over 18 years of age, spoke English as their primary language, were Australian citizens and reported having worked with a mean of 4 ± 2 professional S&C coaches. The profiles of the athletes are summarized in Table 1.

### 2.2. Design

Using the 3+1 C’s model [11] as a framework, and based on contemporary research on coach–athlete relationships and best practice of S&C coaches i.e., [1,6,8], a semi-structured, open-ended interview guide was developed. Open-ended interviews allow the participants to identify a broad range of influential factors that promote both positive and negatively impacted coach–athlete relationships [20]. A deductive thematic analysis was undertaken on the interview transcripts to illustrate portrayed character traits, effective behaviours that display these traits; and how relationships were fostered with athletes by S&C coaches. Specifically, a deductive approach was conducted to remove data not relevant to the pre-identified general dimensions of the 3+1 C’s framework and test its transfer into the context of S&C.

### 2.3. Procedure

**Figure 1:**
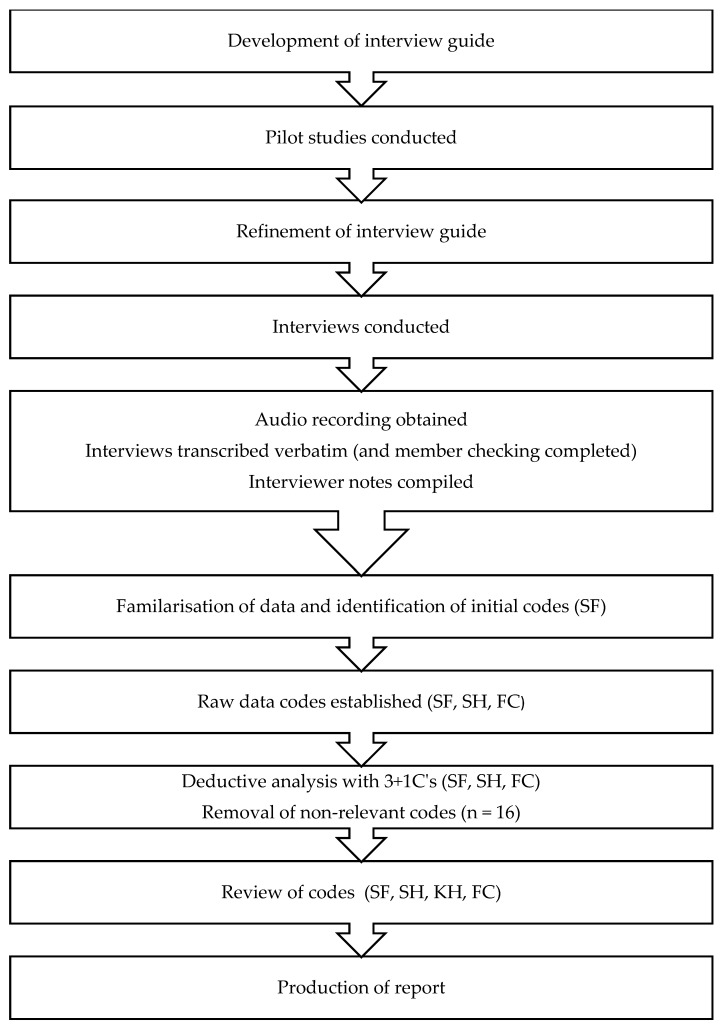
Flow diagram of methods.

A flow diagram of the procedure is provided in Figure 1. To obtain familiarity with the interview process and to ensure that the structure and flow of the interview guide was appropriate, two pilot interviews were conducted. These pilot interviews allowed for consolidating and modification of the interview protocol and provided considerations for conducting the interview [21]. After the pilot interviews, the interview guide was refined in both composition and structure. As a result, the final interview guide included 32 questions covering the following themes:

(1) Background demographic information;
(2) Playing/competitive history;
(3) Details of previous S&C coaches;
(4) Perceptions of S&C coach attributes;
(5) Perceptions of how the S&C coach–athlete relationship was built and maintained;
(6) Perceptions of how S&C coach and athlete behaviours were complimentary;
(7) Perceptions of how alike S&C coach and athlete are/were thinking, feeling, and behaving.

Interviews were conducted face-to-face and recorded verbatim using two hand-held voice recorders (WS-811, Olympus, Hamburg, Germany, 2012) with each interview lasting between 19 and 47 min (mean ± SD: 28:38 ± 7:38 min). Each audio recording was transcribed and then coded using NVivo software (QSR International, Melbourne, Australia, 2017). For confidentiality names of people, places, teams, and organizations were removed from the transcript and replaced with pseudonyms. Participants were also given an opportunity to review the transcript to note any accidental errors of fact, and to remove anything that they did not want included in the final analysis.

Customary with qualitative analysis and following established guidelines [22], trustworthiness of the research was established by the following methods: (1) Credibility—the qualitative equivalent of internal validity was achieved via peer debriefing, negative case analysis, and member checking; (2) Transferability—the qualitative equivalent of external validity was attained by thick description of the procedure; (3) Conceivability—the qualitative equivalent to objectivity was established by developing an audit trail, triangulation of the data, and reflexivity to remove researcher bias; and (4) Dependability—the qualitative equivalent to reliability was enhanced using step-wise replication with the interview guide and an inquiry audit.

### 2.4. Data Analysis

All interviews were recorded in their entirety and transcribed verbatim accounting for a total of 188 pages of single-spaced data. A deductive thematic analysis, based on the “3+1 C’s” framework [11], was used on the interview transcripts following guidelines suggested by Braun and Clarke [23]. The first phase involved familiarization with the data; this required the first author (S.J.F.) to immerse themselves through repeated readings of the transcriptions, re-listening to the recordings, and undertaking a continuous search for codes during analysis. The second phase, conducted by three members of the research team (S.J.F., S.M.H., F.C.) developed an initial list of codes. These codes are raw elements of data most relevant to the research and deeper than themes. Sixteen codes that could not be classified under the 3+1 C themes were deductively removed. These codes were unrelated to the coach–athlete relationship and focused on specifics of the training program (i.e., structure of each session; load management) or previous athletic experience of the coach (i.e., coach’s athletic success). The third phase involved re-reading and reviewing the data collected at code level, with considerations made for coherent patterns within themes, before any codes missed during previous phases were added. The third phase was completed by all members of the research team. The final phase was to produce the report, convincing the reader of the importance of the research, its analysis, and essentially, what it said. Exploration beyond the data was conducted and an argument that relates to the research question was presented.

## 3. Results

From the deductive analysis, 56 raw data themes were identified relating to athletes’ perceptions of effective S&C coach behaviors, which aligned to the 3+1 C’s model. The general dimension of “closeness” covered 26 raw data themes, which combined to form five higher-order themes (trust, like, respect, appreciation, and care). Seven raw data themes were coalesced into three higher order themes (positive outlook, shared experience, and athlete-centered focus) that formed the general dimension of “commitment”. “Complementarity” as a general dimension was developed from 13 raw data themes, from which three higher order themes emerged (adaptability, an autonomy-supportive motivational climate, and role model traits. The final general dimension was “co-orientation”, under which 12 raw data themes were identified and categorized under three higher order themes (teamwork, personality traits, and effective communication). This data is represented in Table 2.

### 3.1. Closeness

Closeness included the higher-order themes of “trust”, “like”, “respect” and “appreciation” as defined by previous literature [11] and it was further found “care” helped develop closeness. These high-order themes were identified from 25 raw data themes.

All participants mentioned that trust was built over time, with preferences for one-on-one scenarios, particularly during return-to-play following injury. Athlete 2 stated, *“are we in a phase… where we want to push something, or do we need to pare it back? and, so being able to have those discussions one on one is critical for me”.* Athlete 6 spoke of their return-to-play scenario: *“I knew he was a good rehab fellow… you see the results... you sort of learn to trust them more... you do another test and you’ve dropped 15 seconds... you know it works”*. In this athlete’s example, trust was further developed in response to seeing success in their program (i.e., getting faster).

Athletes desired a mateship with coaches but acknowledged concerns with professionalism and boundaries. *“You want to be mates … but at the end of the day… they’ve got to tell you... what to do… they can’t be too much”* (Athlete 6). Similarly, athletes’ perception of respect from their coach was characterized by outward displays to improve the athlete and caring about the athletes’ success, active listening and time management. Athlete 4 described how their S&C coach expressed their desire to improve the athlete with *“constant conversation …what can we do today to make you a better athlete and to get you to where you need to be?”* Further, athletes also felt valued by the coach’s punctuality and time keeping.

The coach’s personal achievements, either witnessed or by reputation, helped build credibility, particularly when the athlete was not involved in the coach’s recruitment. Athlete 1 noted *“…just (COACH)’s accolades and who (COACH) is working with and what kind of results (COACH) has achieved … that’s enough for me”*. Most athletes responded almost identically. Athlete 4 employs their own coach and noted reputation forms part of their selection criteria: *“I do my due diligence before I work with anyone… and that process usually involves knowing their past history.”*

During coaching sessions, the acknowledgment of an athlete’s effort (e.g., “good lift”) and follow-up contact were perceived as positive characteristics. Athlete 10 shared their S&C coach *“just genuinely might send me a message… like, Okay, how is it going? How did nationals go?”* and valued the coach’s recognition on social media *“(COACH) put social media posts up… in support of me”*. Further, conversations beyond sport and training (i.e., about children), were found to be very important to the athletes. Athlete 3 highly valued having *“been able to go to them with… anything else that I might have on my plate mentally as well, and be able to talk to them about those”*.

### 3.2. Commitment

Commitment was identified by three higher order themes; positive outlook, shared experience, and an athlete-centered mindset. A positive outlook from the S&C coach was perceived as showing a long-term commitment and was displayed by future planning, mutual goal setting, and a focused work ethic. Athlete 8 perceived future planning with their coach as a personal investment, sharing an example conversation; *“This is what we did last year, this is what you were going to do this year, this is where we wanted you to be at this point in time”*. Coach–athlete relationships were built on prolonged engagement, where time spent in each other’s company was essential to the relationship. Athlete 6 explained that their journey with their S&C coach began through injury rehabilitation and had developed over six years during both positive and negative times, where the coach provided essential support. Furthermore, Athlete 4 stated:

“the support is constantly there and it doesn’t really matter… what time of the year it is, what phase of training you’re in, whether things are going wrong or bad or if there are… circumstances that are going to affect you outside of your control, they’re still giving you the same amount of time and energy… they’re there for you, and then they’re pushing to get you to where you need to go, in spite of the complexity around you.”

### 3.3. Complementarity

Complementarity covered the higher-order themes of adaptability, an autonomy-supportive motivational climate and the coach’s role model traits. Athletes reported preferences for a coach who displayed adaptability regarding training variations, coordinating schedules, and adjusting loads based on feedback. Athlete 2 shared how they discussed training session variables such as days, length, and exercise preferences, and their S&C coach then developed a program accordingly. Athlete 10 explained how their coach adapted their sessions based on what was available during international travel. Coaches were also able to adapt training depending on how the athlete reported feeling. Athlete 8 discussed the importance of being able to adapt spontaneously

“being able to have a good relationship and for them to be able to read... how the athlete or the group is feeling, whether that is… pushing them harder or taking it easy some days… not necessarily…feeling like you need to follow the program exactly.”

Athletes described how their S&C coaches created an autonomy-supportive motivational climate, with Athlete 8 stating the best S&C coaches are *“that loud, spark and positive energy straight away especially in morning sessions”* and *“just being in that S&C environment is pretty motivating in itself… with everyone else training here as well”.* Athlete 10 noted it was not just their coach’s actions, but the timing of their actions; *“(COACH) chooses the right time to be loud”*. Displays of passion from the S&C coach also appeared to motivate some athletes. Athlete 4 provided the example

“…the passion they have for helping you get to where you’re going… if they’re into what they’re doing, if they’re excited by the training, if they’re excited by the progressions that are being made… when… push comes to shove, they love what they do and – and strength and conditioning is at the heart of what they want. So they’re excited to be there and they’re excited to work with you.”

Athletes mentioned observing their coach training themselves demonstrated significant role model characteristics. Athletes shared they felt seeing their coach train was setting a good example. Athlete 3 viewed their coach behaving in a consistent manner as an important role model trait, stating they *“will always be there… always show up with the same … attitude”.* Being accountable and *“leading through example… is the most powerful form of effective leadership”* (Athlete 4). 

### 3.4. Co-orientation

Co-orientation, encompassed three higher-order themes; teamwork, personality traits, and effective communication. Teamwork was developed by how the coach acted on athletes’ suggestions and reciprocal accountability. Athlete 4 described how they felt about a shared purpose as *“the biggest thing is that you have a shared passion, a shared purpose, and… if those two are in alignment, then it’s a pretty easy thing… to have connection and to support one another.”*

Three athletes articulated similar feelings of the team working towards the same goal, with Athlete 7 describing their specific shared purpose as working on team values, while individually pursuing Olympic gold. Athlete 3 elaborated:

“there’s a massive difference between listening to understand and listening to responding; … my favorite coach has always listened to try and help me figure out a situation, rather than be: “I have a problem. You have to come up with a solution”, or “you’re the problem”… it’s not - it’s us working together”.

Humor was recognized as a preferred personality trait of S&C coaches. Athlete 1 discussed humor as making *“the whole environment a bit more enjoyable”*. This was a common thread across all athletes. Having a holistic view to health was also a favored trait. Specifically, Athlete 2 said they shared with their S&C coach: *“a core belief of health and wellness above fitness because a lot of people are fit but they are not healthy… I think an important aspect is… focus on mental health as well.”*

Other athletes spoke of how the S&C coach had referred them to a mental health care specialist for further support, and that this was considered a critical step in the preparation for career termination. *“They would like to transition me out of being an elite athlete into just being a healthy, functional human… we are focusing mainly on long term”* (Athlete 12).

Effective communication between the athlete and S&C coach was developed from direct feedback, openness, and individualizing coaching style. Athlete 11 said *“I prefer when a coach is direct with me and just tells me, okay you need to work on this, you need to do this better”*. Athlete 2 spoke of openness between the coach and athlete being effective; saying *“I could say anything about the training and be totally open”* and further adding *“being able to communicate freely… if you think something is not right, being able to express that.”* Athlete 3 shared how they’d evolved over their career:

“I never used to think like this as a younger athlete, but I definitely do now—being very open… how you want the relationship to work, because they’re not all just going to form naturally. I think it is actually really important to discuss how you want it to look like… that should be a goal.”

Athletes emphasized an importance on individualized communication, saying “*main one for (COACH) would definitely be communication over everything; the way that (COACH) is able to alter the way (COACH) communicates to individuals*” (Athlete 3). Athlete 11 shared a similar observation; “*it’s important that the coach understands the player and how they respond to criticism and feedback as well*”.

## 4. Discussion

The purpose of this project was to investigate high performance athletes’ perceptions of S&C coaches, specifically, (1) their character traits; (2) their effective behaviours that display these traits; and (3) how coach–athlete relationships were fostered. The results support previous research that highlighted the effectiveness of instruction and technical knowledge [24,25], but further recognizes the importance of transferable skills to the S&C coach [18]. The findings of the current study supports the relationship being dyadic [12]. Athletes preferenced coaches who had a positive outlook and an athlete-centered mindset, with active listening and individualized goal setting considered key features to developing a positive relationship.

Openness and honesty are central to building positive coach–athlete relationships [26]. The use of humor has been linked to how well liked the coach is, enhanced communication, and indirectly may improve performance [27]. The ability of the S&C coach to engage with the athlete on a personal level developed closeness and trust within the relationship, which enabled more positive outcomes for both the relationship and the athlete’s performance [3]. Of interest was the importance each athlete placed on their coach’s ability to take an interest in what they do beyond the sporting environment, as this demonstrated a display of care on behalf of the coach.

S&C coaches are encouraged to observe the athlete’s responses to identify if it is necessary to make changes to their communication style, behaviour, or action to increase the likelihood of achieving the desired outcome [17]. Athlete responses in the current study highlight the importance of adaptability within the coaching approach and the need to individualize training programs. Accurate and timely feedback is required to improve performance, but considerations need to be made for how it is delivered, with concise, constructive feedback preferred [18]. The use of critical self-reflection on practice has been regularly implemented in general coaching practice as a means to improve coach effectiveness [6]. 

Participants frequently used the term “attentive” as a preferred trait, however previous literature has not used the term. Nevertheless, participants viewed individual program design as a sign of the coach being attentive and preferenced this behaviour. No research has been conducted on this link, although there is support suggesting that individualized programs are significantly more effective for performance outcomes [28].

Coach–athlete relationships are built over time, with prolonged engagement being advantageous for positive relations [29]. Success in a coach–athlete relationship was possible where they work together toward one goal i.e., a “shared purpose”, with athletes in the present study highly valuing the mutual goal setting process. Athletes go through positive and negative experiences and periods, and the S&C coach is in a position to provide both problem-focused and emotional support. Szedlak, Smith, Day, and Greenlees [18] reported athletes positively perceived coaches who provided encouragement, inspiration, and reassurance, and the responses from the athletes in this study support this.

## 5. Conclusions

The present study is the first, to our knowledge, to examine high performance athletes’ perceptions of S&C coaches within Australia. The findings highlight the importance of building of positive coach–athlete relationship for the athlete, with transferable skills integral to this. With this in mind, we propose that S&C coach education should not just focus on the prescribable aspects (i.e., technical skills, recovery strategies) but also incorporate transferable skills and guidelines to develop positive relationships. General sport coaching literature recognizes athlete’s perception of coach behaviours effect sporting outcomes and the current study supports this in S&C settings. Athletes preferred coaches who have a positive outlook and an athlete-centered mindset. The ability to work with the athlete as an individual, setting mutual goals, and adapting training programs to specific needs was important for building trust in the relationship. Findings also note the importance of taking a holistic approach to the athlete’s development and welfare, and encourage S&C coaches to take an interest in their athletes’ life outside of the sporting arena.

## Figures and Tables

**Table 1:** Descriptive characteristics of athletes.

Athlete	Age (Years)	Sex	Sport Most Recently Competed in	Number of Previous S&C Coaches	Average Weekly Contact Hours with S&C Coach
1	37	Male	Freestyle Wrestling *	2	2
2	53	Male	Triathlon *	1	2
3	25	Female	Field hockey	6	3
4	32	Female	Cycling *	8	6
5	26	Female	Rowing *	6	3
6	37	Male	Rugby Union	5	5
7	20	Male	Cycling *	2	3
8	24	Female	Netball	3	2
9	20	Male	Rugby Union	3	5
10	28	Female	Table Tennis *	5	2
11	18	Male	Rugby Union	3	5
12	30	Female	Ice Hockey	5	2

* Athlete employed their own or worked with an Institute of Sport-provided S&C coach. All others worked with an S&C coach employed as part of a wider organisational coaching team.

**Table 2:**
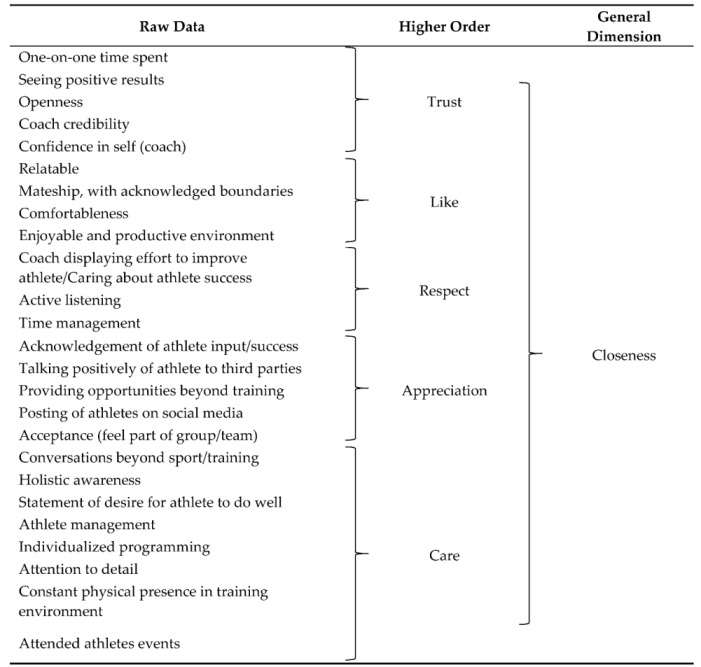

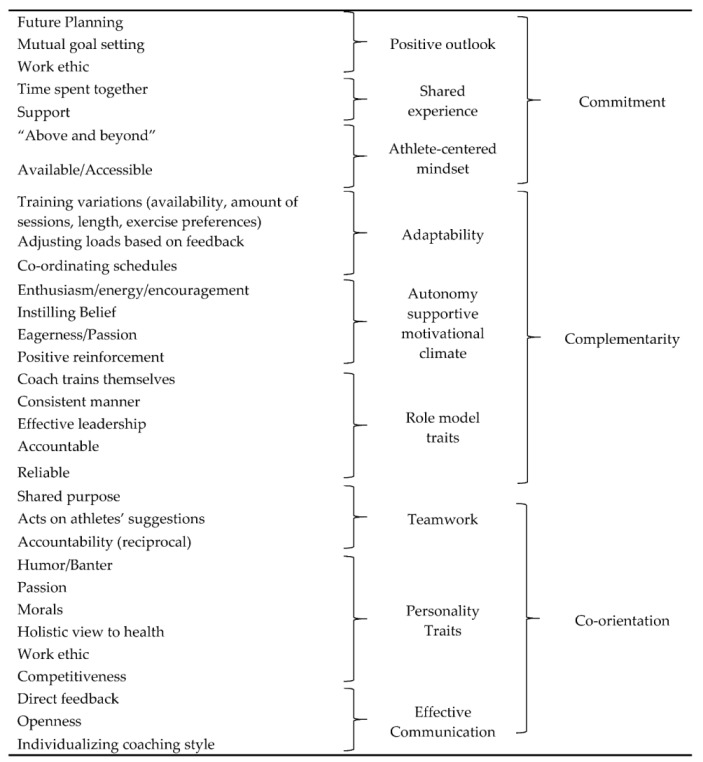
Athletes’ perceptions of effective S&C coach behaviours within the 3+1 C’s model.
